# New Appliances and Things Medical

**Published:** 1910-10-01

**Authors:** 


### NEW APPLIANCES AND THINGS MEDICAL.

We shall be glad to receive at our Office, 23 & 29 Southampton Street, Strand, London, W.C., from the manufacturers, specimens of all new preparations and appliances.]

### NAZENE AND THE NASAL BATH.

(Nazene Co., 30 and 31 Great Marlborough Street, W.)

Nazene is an amorphous white powder, which makes a solution in many respects analogous to the lotio alkalina of the hospital pharmacopœias. It dissolves easily in lukewarm water, is non-poisonous and non-irritating, and used with the nasal bath—a simple china receptacle which fits fairly well to the nostrils and seems admirably suited to its purpose—it supplies an excellent douching medium. So used it is remarkably soothing, and we can quite understand that it will be of immense benefit to those whose nasal condition is such that they need a periodical douching. In cases of adenoids and enlarged inferior turbinals, septal deformities, especially in children, and the many pathological conditions met with in the nose, Nazene can be confidently recommended, although we do not consider it superior to the ordinary flushing media when used with similar discrimination and perseverence. The nasal bath deserves to be widely known. It is one of the best models we have seen.

### CARNAVITIS TONIC WINE.

(P. B. King & Co., Ltd., St. Thomas Street, Portsmouth.)

There are many varieties of "tonic wines" on the market, some of which are of no value at all from a dietetic point of view, while others are certainly of undoubted use as general stimulating tonics. Among the latter Carnavitis should occupy a high place. It is practically a solution of meat extract, with which is incorporated a malto-dextrose of decided starch-splitting power, in a port wine basis. The amount of sugar it contains is somewhat high for such a wine, but on the whole Carnavitis is pleasant to the taste and quite free from the druggy flavour which characterises so many vinous meat extracts. As an appetiser and tonic it is of undoubted value, and although its iron content is small it is still appreciable. We have found the samples submitted to us to possess decided stimulating properties, though the percentage of alcohol is not greater than is to be found in a good sound wine with a fair amount of body such as is the basis of Carnavitis. The combination is well worth a trial in all cases in which the exhibition of such a stimulant is considered necessary. Sample bottles and literature are sent free on application to the manufacturers, whose address is given above.

### MACHINERY SPONGE CLOTHS.

(Albert Durst and Frankl, 65 Coburg Road, Old Kent Road, London, S.E.)

The machine cloths manufactured by this firm are of a durable quality which wears well, and are quite washable. They are manufactured in large pieces, measuring 19 in. by 20 in., and sold at 24s. per gross net cash. The firm undertakes the washing of these cloths at the rate of 2s. 6d. per hundred, which price not only includes all cartage in London and district, if not less than a hundred cloths at a time are returned, but also includes the replacing of all unserviceable cloths free of charge for one year, so that the cost of wear and tear falls not on the institution, but upon the firm. The firm also loans cloths on certain conditions, and is prepared to make quotations for muslin and butter cloths at certain special institutional rates. The cloths turned out by this house are excellent in every respect, and fully merit the attention of hospital secretaries and others who are interested in coverings for meat safes, cleaning cloths, and similar textiles.

### THE EUREKA CREPE VELPEAU BANDAGE.

(Vincent Wood, Surgical-Appliance Maker, Victoria House, Albion Place, Blackfriars Bridge, S.E.)

The Eureka Crêpe Velpeau bandage is particularly interesting as it contains absolutely no rubber, but is highly elastic, cool, porous, and easily washable, and therefore economical and lasting. The prices charged by this firm are very low in comparison with the superfine quality of goods turned out, and the specimen bandage submitted to us is certainly one of the best of its kind that we remember having seen. It is a well-known fact that crêpe velpeau bandages are especially suitable for the treatment of varicose ulcers, but that the ordinary qualities are both expensive and flimsy in texture, hardly retaining their elasticity as they ought to do. The "Eureka," however, is of an entirely different type. The sample sent to us is of the best quality, very light, very easily adjustable, as a velpeau bandage ought to be, and porous enough to permit of discharges oozing through. We have for some time used the firm's "second quality" (blue line) bandages in the treatment of sprains, and have found them excellent in every way and slightly cheaper than the "red line" qualities. The firm also manufactures special crêpe velpeau abdominal binders which well deserve a trial. It is important that buyers should see that the name "Eureka" is on the wrapper, as there are many imitations on the market which are distinctly inferior, and the use of which will only lead to dissatisfaction. Quotations and full particulars may be obtained on application to the firm at the above address.

### THE DOCTOR'S HOUSE.

(Building and Estates Development Company, Limited, Bristol.)

The country doctor often experiences a difficulty in finding a house of the accommodation he requires and at such a price as a small practice will warrant. We have had a plan sent to us of a detached villa that should quite meet his requirements in both respects. It contains an entrance hall with waiting-room and surgery adjoining, drawing-room, dining-room, large kitchen with offices on the ground floor, and four bedrooms, bathroom and w.c. on the first floor. This is the most compact house we have seen, and the pretty entrance porch makes the elevation a very pleasing one. This villa, which is built on Calway's patent reinforced monolithic concrete wall system with both vertical and longitudinal air-cavities in the thickness of the wall, which thoroughly insulate the house and provide dry walls, can be erected complete and ready for occupation for approximately £450. If desired, the contractors can, as a rule, leave two-thirds of this amount on mortgage. The Building and Estates Development Company, Limited, of Bristol, will be pleased to give any further information, or for 1s. will send a printed plan of this house. Houses of any size or for any purpose are, of course, built by the company, including infectious disease and other hospitals, and one has only to mention his requirements to be speedily suited. The illustrated booklet issued by the firm gives full particulars about the various specialities, and will well repay study.

